# Innovative dead-time correction and background subtraction for neutron multiplicity measurements using neural networks

**DOI:** 10.1038/s41598-024-57778-5

**Published:** 2024-03-30

**Authors:** Jeremias Garcia-Duarte, Yonatan Mishnayot, Aaron S. Tamashiro, Jackson R. Lawrence, Jason T. Harke

**Affiliations:** Lawrence Livermore National Laboratory, NACS, Livermore, CA 94550 USA

**Keywords:** Experimental nuclear physics, Characterization and analytical techniques

## Abstract

The number of neutrons emitted from a nuclear reaction plays a crucial role in various fields, including nuclear theory, nuclear nonproliferation, nuclear energy and nuclear criticality safety. Accurate determination of neutron multiplicities requires the application of several corrections, with dead-time correction and background subtraction being particularly significant. These corrections become more challenging for neutron detectors with time-dependent neutron capture. In this work, we perform a comprehensive study of three existing methods used for dead-time correction and background subtraction in neutron detectors with time-dependent neutron capture. The methods were tested for dead-times in the range from 0 to 1 μs using a Monte Carlo model simulating the dead-time and background effects in the standard neutron multiplicity probability distribution of $^{252}$Cf. The previous methods showed larger than desired uncertainty or systematic trade off. Those uncertainties prompted the development of a novel approach using neural networks trained with data from Monte Carlo simulations. The Neural Network method enabled the correction of neutron multiplicity probabilities more accurately than the other methods with fractional errors smaller than 3% for multiplicities around the peak of $^{252}$Cf. A similar approach using neural networks could be applied to problems where the system being studied can be accurately simulated without having an accurate analytical description available. The neural network method presented in this paper can be easily expanded if multiplicities greater than 10 are expected.

## Introduction

The development of the first large liquid scintillator counter for neutron detection at the Los Alamos National Laboratory (LANL) made it possible to measure not only the average number of emitted neutrons but also the neutron multiplicity probabilities^1^, i.e. the probability of emitting *n* neutrons ($D_n$). The first experiments using this detector to measure the neutron multiplicity probability distribution of various fissioning nuclides were reported by Diven^2^ and Hopkins^3^. In these experiments, a dead-time correction was introduced to account for a single pulse overlap, i.e. two events with a time difference smaller than the dead-time per event of the data acquisition electronics, which in their case was $\tau$ = 0.15 μs. The effect of the background on the neutron multiplicity distribution was then corrected by solving a system of equations. The final neutron multiplicity distribution was obtained after correcting for the detector efficiency. However, as later found by Moat^4^, the validity of Diven’s analytical method holds only for a small concentration of the neutron capture element loaded in the scintillator, low backgrounds and short dead-times. The larger the concentrations of neutron capture element loaded in the scintillator, the shorter the time window where neutrons are absorbed, increasing the overlap probability. Moat^4^ conducted a similar experiment with a dead-time of $\tau$ = 0.25 μs using their own simulation method to overcome the limitations of Diven’s analytical method. In a second paper by the same group, Mather^5^ clarified that a Monte Carlo (MC) model was used to obtain a matrix that transforms the experimental into the real neutron multiplicity probability distribution. A different formulation of the MC method was later introduced by Ribrag^6^ where the background correction was performed with an analytical method and the dead-time correction with a MC method similar to Moat’s method. In the work reported by Jahnke^7^, each multiplicity is corrected individually using an average dead-time correction obtained from MC simulations.

The first to develop an analytical method to simultaneously correct for dead-time and background was Boldeman^8,9^, where only a single overlap per detection gate was considered since the measured dead-time was small ($\tau$ = 0.076 μs). The detection gate is defined as the time interval after the trigger event occurs where the acquisition channels of the electronics are open waiting for an event to be recorded. Diven’s method was improved by Frehaut^10^, who included more than one overlap per detection gate in (n, 2n) and (n, 3n) cross section measurements. Frehaut applied the background correction introduced by Baron^11^ and then used an improved version of Diven’s method^2^ for dead-time correction. The time distribution of neutron capture in liquid scintillators is a crucial part of the analytical methods for dead-time correction. Parker^12^ explored in depth the parameters affecting the time distribution of neutron capture events in liquid scintillators for (n, 2n) cross section measurements. A different formulation of the analytical method was introduced by Spencer^13^ where the background and dead-time corrections are performed separately. First the background is unfolded by solving a system of equations. Spencer^14^ initially used Frehaut’s version^10^ of Diven’s method^2^ to correct for the dead-time overlap and unfold the background. However, due to the limitations of these methods, Spencer^13^ introduced a different formulation for dead-time correction following the work of Ribrag^6^. The dead-time correction in Spencer’s method involves several integrals to calculate each element of the correction matrix and then solve the resulting system of equations.

The next generation of large liquid scintillator detectors designed for neutron multiplicity measurements exhibit longer electronics dead-times due to the use of digitizers for data acquisition, with $\tau$ = 0.3 μs for the detector used by Dushin^15^ and $\tau$ = 0.9 μs for the NeutronSTARS detector used by Akindele^16^. As anticipated, the analytical method proposed by Diven^2^ is inadequate when dealing with a dead-time of $\tau$ = 0.9 μs. However, Dushin^15^ successfully employed the MC method introduced by Moat^4^ to perform the dead-time correction for $\tau$ = 0.3 μs. Ideally, the electronics dead-time should be kept as short as possible. In the case of NeutronSTARS, the expected dead-time from vendor’s specifications was around 0.120 μs. However, the measured dead-time was found to be much longer, on the order of 0.9 μs.

Inevitably, we are faced with the questions of whether those methods present accurate results for dead-times as long as $\tau$ = 1.0 μs, and if not, what is the maximum dead-time threshold where the analytical methods and the MC method begin to fail. Additionally, we aim to explore the feasibility of an alternative approach to correct the neutron multiplicity probability distribution impacted by dead-time and background effects. This paper provides a comprehensive overview of three methods used for dead-time correction and background subtraction and introduces a promising alternative in the form of a MC-trained neural network (NN). The NN is trained using data generated from a MC simulation based on the characteristics of the NeutronSTARS large liquid scintillator detector^16^. The NeutronSTARS detector is used as a comparison reference in this manuscript. However, the results here presented are applicable to all detectors with a time dependent event distribution in the detection gate like for example the BF$_3$ detector assembly from the work of Lees^20^.

## Results and discussion

In this study, we delved into the complexities of dead-time correction and background subtraction methods used in neutron multiplicity measurements. We conducted Monte Carlo simulations to evaluate the impact of dead-time overlaps in the case of neutron detectors with time-dependent neutron capture. Using the $^{252}$Cf standard neutron multiplicity distribution from the work of Santi^17^ and the characteristics of the NeutronSTARS array^16^, our investigations revealed that at a dead-time of $\tau = 1 \, \upmu s$ nearly 29% of triggers resulted in the loss of one neutron, while 5% lost two, underscoring the critical importance of using an accurate dead-time correction technique (Fig. 2).

**Figure 1:**
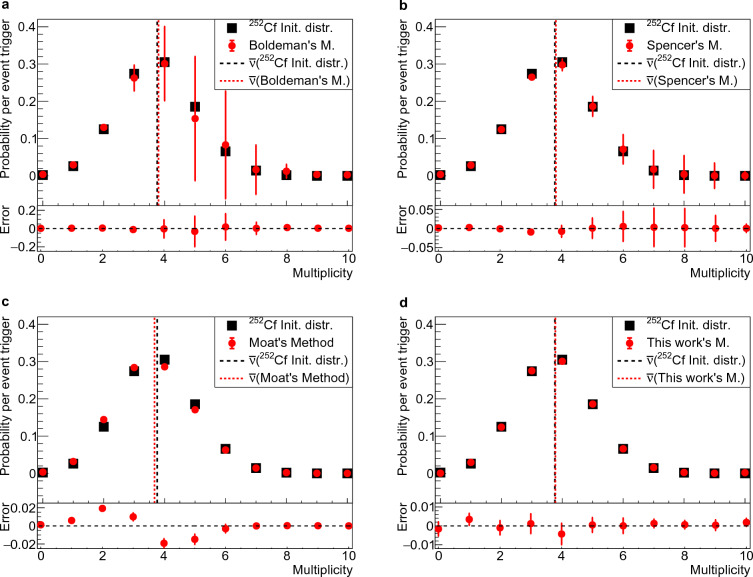
Comparing correction methods at 0.9 $\upmu$s dead-time. The standard $^{252}$Cf multiplicity probability distribution^17^ was used to sample the multiplicity 10$^5$ times in a detection gate of 60$\upmu$s duration, with time distribution and background rate from the NeutronSTARS detector^16^. The MC simulation was repeated to generate the simulated observed data which was then corrected using four different methods. Each plot contains the initial $^{252}$Cf multiplicity distribution in black squares and the results in red circles, with an underneath plot containing the error of each method: **a** Boldeman’s; **b** Spencer’s; **c** Moat’s; and, **d** this work’s method. The error bars are the standard deviation of the results from the many MC repetitions. The dashed (Initial distribution) and dotted lines (Results) are the average number of neutrons emitted per detection gate ${\bar{\nu }}$.

Upon scrutinizing Boldeman’s analytical method^9^, limitations surfaced particularly from $\tau = 0.2 \, \upmu s$ onward. Through extensive iterations, a significant degeneracy was revealed, rendering Boldeman’s analytical method impractical for dead-times approaching a few hundred nanoseconds^4^. This degeneracy, vividly illustrated in Fig. 3, led to substantial uncertainties from a dead-time of $\tau = 0.2 \, \upmu s$ and higher, where absolute errors exceeded 0.4 for all multiplicities, resulting in fractional errors of more than 100%. The standard deviation of the errors for multiplicities 4, 5 and 6 is more than 0.1 at $\tau = 0.9 \, \upmu s$ (Fig. 1), equivalent to fractional errors ranging from 30% to more than 100% respectively. We also tested the analytical method introduced by Spencer^13^. Spencer’s integral method presents a smaller degeneracy and the standard deviation of the errors for multiplicities 4, 5 and 6 exceeds 0.02 at $\tau = 0.9 \, \upmu s$ (Fig. 1), equivalent to fractional errors ranging from 7% to around 29% respectively.

Our exploration extended to Moat’s MC method, unveiling a linear growth in errors with increasing dead-times. Although free from degeneracy, this method introduced fractional errors of around 15% for multiplicity 2 and 7% for multiplicity 4 at $\tau = 0.9 \, \upmu s$ (Fig. 1). It also presented the largest error in the average number of neutrons per detection gate ${\bar{\nu }}$, around 2.4%. Embracing advanced computational techniques, the NN approach emerged as a promising method for enhanced accuracy. Trained on MC data based on the NeutronSTARS characteristics, the NN method demonstrated exceptional resilience with mean errors consistently below 0.005 at $\tau = 0.9 \, \upmu s$ (Fig. 1), equivalent to fractional errors smaller than 2% for multiplicities 3, 4 and 5. This approach also presented the smallest error in the average number of neutrons per detection gate, showcasing its potential in enhancing the accuracy of neutron multiplicity measurements.

In the subsequent sections, we delve deeper into each aspect of our findings, elucidating the complexities of dead-time overlap effects, the limitations of currently available methods, and the benefits of the innovative MC-trained NN method. The results refine our understanding of the corrections involved in neutron multiplicity measurements, offering valuable insights for future experimental endeavors.

### Dead-time overlap effect

In this work we only consider systems of the “nonparalyzable” type, that is a pulse occurring during the dead-time of the previous one does not introduce additional dead-time. In the past six years our group performed several experiments using the large liquid scintillator detector array NeutronSTARS^16^. A MC model was developed in C++ to gain insight into the number of lost events resulting from dead-time overlaps using this detector.

**Figure 2:**
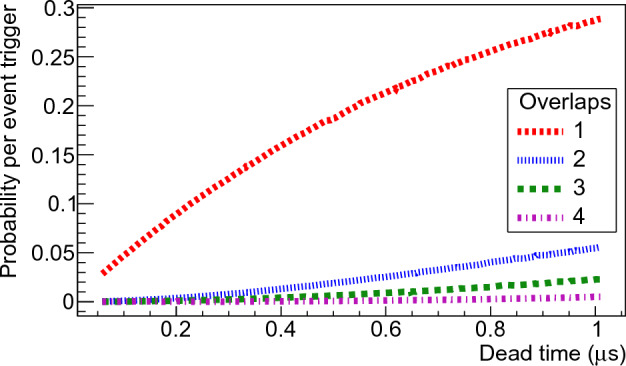
Dead-time overlap effect. The probability per detection gate of one or more overlaps were simulated for different dead-times. The standard $^{252}$Cf multiplicity probability distribution^17^ was used to sample the multiplicity in a detection gate of 60$\upmu$s duration, with time distribution and background rate from the NeutronSTARS detector^16^. The results only include neutron-neutron and neutron-background overlaps since we are interested in how many real events are lost.

Trigger events were generated, and for each trigger the multiplicity was sampled from the standard multiplicity probability distribution of $^{252}$Cf reported by Santi^17^. Random seeds were utilized to initialize all random variates. The neutron multiplicity was generated using the inverse transform method, while the time of each event was generated using the Accept-Reject method^18^. The time of each neutron event was sampled from the time distribution shown in Fig. 8, and a detection efficiency of 100% was used. As shown in Eqs. (9), (10) and (11), the dead-time overlap probability do not depend on the detection efficiency. A lower efficiency would shift the multiplicity distribution towards lower multiplicities reducing the effect of dead-time overlap. For each trigger the multiplicity and time of the background events were sampled considering a typical rate for NeutronSTARS of 20 kHz in a detection window of 60 $\upmu$s. Then, a time difference matrix was generated for each given trigger with the time difference between all available neutron and background events. The dead-time overlap followed a simple selection rule where if the time difference is smaller than $\tau$ = 0.9 $\upmu$s, the event was rejected and the number of overlaps incremented. Only neutron-neutron and neutron-background overlaps were included in the overlap probability calculation since the background-background overlap does not contribute to the loss of real neutron events. A distinction is made between a reaction or fission neutron event, and a background event because they have different time distributions within the detection gate. Figure 2 shows the probability per trigger event of up to 4 overlaps for dead-times up to 1 $\upmu$s. One overlap means that one neutron was lost during the detection gate, and four overlaps means that four neutrons were lost during the detection gate. At $\tau$ = 1 $\upmu$s almost 29% of the triggers lose one neutron and 5% lose two neutrons for the $^{252}$Cf neutron multiplicity distribution. Different from what was assumed by Lott^19^, for systems of the non-paralyzable type, the dead-time overlap probability does not depend on the reaction rate. The dead-time overlap probability depends on three factors: the dead-time per event of the acquisition electronics, the duration of the detection window, and the neutron capture time distribution of the detector.

### Boldeman’s analytical method

Boldeman’s method is described in the Methods section “Boldeman’s analytical method”. The implementation of this method and the MC model used to test it are described in the Methods section “Implementation of Boldeman’s method” and “Monte Carlo model to test Boldeman’s method’. Boldeman’s method was tested with a MC-generated data that simulates the observed data for the $^{252}$Cf standard distribution^17^. We found that, for each MC example we obtained a very different output from the solver developed for Boldeman’s method. After repeating this process several times we obtained a Gaussian probability distribution for each multiplicity that should reflect the statistical variation of the MC simulation. The standard deviation and mean values for each multiplicity are shown in Fig. 1 in comparison with the true multiplicity probability distribution. The results clearly show a degeneracy behavior much larger than the expected statistical variation from the MC simulation which is repeated 10$^5$ times. This observation is consistent with Moat’s findings^4^, where it was emphasized that Diven’s analytical method, a purely analytical method like Boldeman’s, becomes impractical when dealing with dead-times larger than a few hundred nanoseconds.

**Figure 3:**
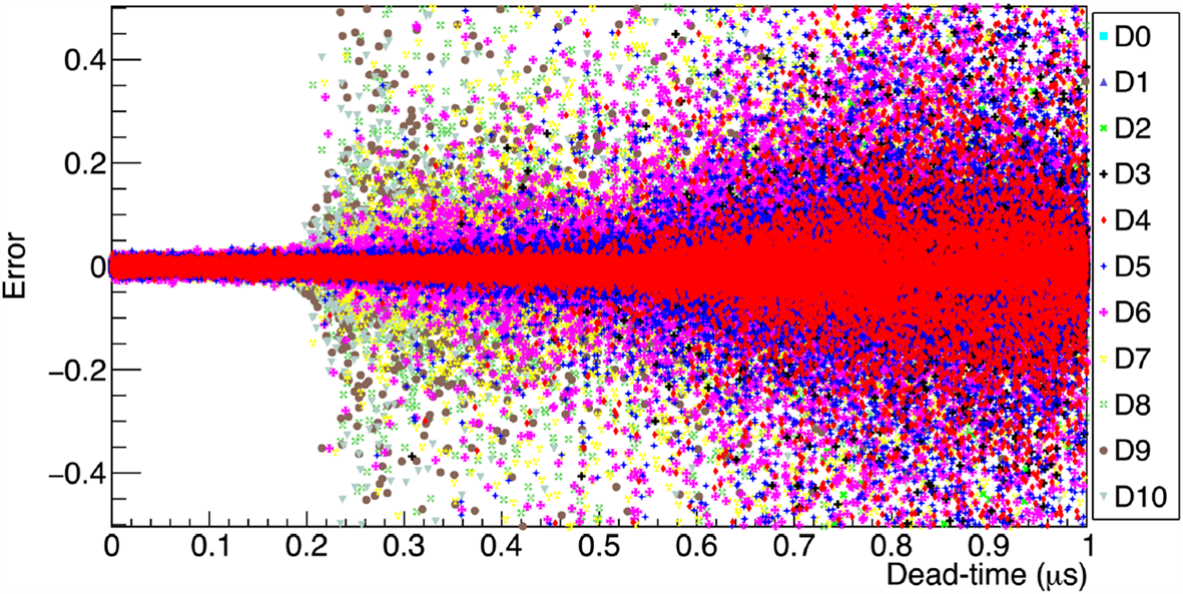
Degeneracy of results from Boldeman’s method. The errors with respect to the initial multiplicity probability values are displayed for several MC repetitions for multiplicities ranging from 0 to 10. For more details see Fig. 1.

We conducted additional simulations varying the dead-time values in the range of 0 to 1 $\upmu$s to identify the point at which Boldeman’s method exhibits degeneracy. We recorded the error of each multiplicity defined as the difference between the calculated value and the true value. The results indicate that at an approximate dead-time of $\tau$ = 0.2 $\upmu$s, certain multiplicities become highly uncertain, with absolute errors exceeding 0.4, equivalent to fractional errors of more than 100% for all multiplicities.

**Figure 4:**
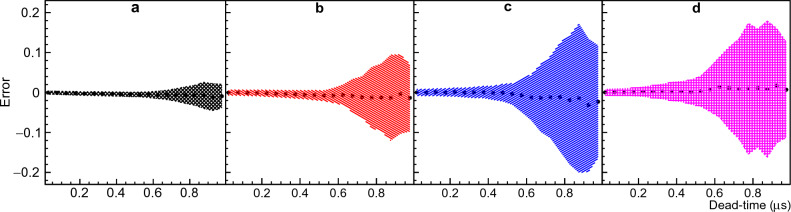
Errors from Boldeman’s method. Figure 3 was divided in 20 dead-time bins, the mean value and standard deviation resulting from the Gaussian fit of the Y-axis projection for each dead-time bin was plotted. Panels (**a**), (**b**), (**c**), and (**d**) correspond to neutron multiplicities 3, 4, 5, and 6, respectively, providing a comprehensive view of the data’s variability. For more details please refer to text.

The scatter plot in Fig. 3 provides a visual representation of the point at which the solver’s results start to exhibit degeneracy. However, it does not provide specific quantitative error values for each multiplicity. To gain a deeper understanding of the behavior of each multiplicity within the context of a standard $^{252}$Cf multiplicity distribution, we divided the X-axis into 20 equally sized bins of 0.05$\upmu$s each and used a Gaussian function to fit the projection along the Y-axis for each dead-time bin. The standard deviation and mean value of the errors were plotted as a function of the dead-time bin center for multiplicities ranging from 3 to 6 in Fig. 4. To obtain a clear picture of this result, it is important to also consider the fractional error defined as the ratio between the error and the true value, which for multiplicities 3, 4, 5 and 6 is around ± 15%, ± 33%, ± 105%, and ± 230% respectively at $\tau$ = 0.9 $\upmu$s.

The analysis described in this section was also performed using single overlap equations (6) and (7) instead of double overlap equations (12) and (13). The results shown here in Figs. 1, 3 and 4 remained unchanged, which demonstrates that the degeneracy observed is not due to the double overlap terms added in equations 12 and 13 but rather due to inaccuracies in the single overlap terms. It is not clear why the mathematical solution for the system of equations become so degenerate. There is clearly a statistical variation in the simulated observed multiplicity probabilities due to the limited number of stories, 10$^{5}$. However, we can infer that this analytical method do not reflect the complexity of the system for longer dead-times when more terms become important for the solution.

It is crucial to highlight the distinction: while our analysis employs MC simulations to generate multiple datasets for a given input multiplicity distribution, an actual experiment yields just one dataset per input multiplicity distribution. When applying Boldeman’s analytical method to this singular dataset from a real experiment, there is a 68% probability that the solution obtained will fall within the error bars depicted in Fig. 1, leading to an unacceptably large uncertainty.

The errors observed in Fig. 1 generate a 1.14% mean error in the average number of neutrons per detection gate calculated with ${\bar{\nu }}=\sum n D_n$. The average number of neutrons emitted per detection gate is 3.757 for $^{252}$Cf from the work of Santi^17^ and 3.80(3) if using Boldeman’s method. This result indicates that the degeneracy observed in Boldeman’s method has a small effect in ${\bar{\nu }}$, and the errors somehow compensate each other when calculating ${\bar{\nu }}$.

### Spencer’s integral method

**Figure 5:**
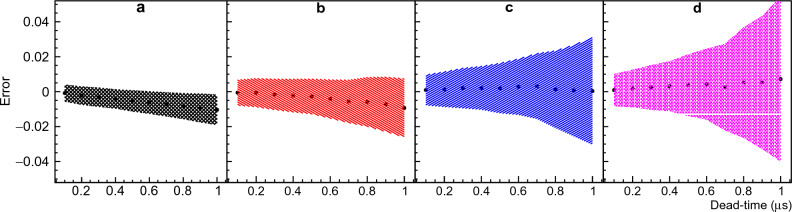
Errors from Spencer’s method. The mean value and standard deviation resulting from the Gaussian fit of the error distribution for ten different dead-times was plotted. Panels (**a**), (**b**), (**c**), and (**d**) correspond to neutron multiplicities 3, 4, 5, and 6, respectively. For more details please refer to text.

After correcting for the background, the MC-generated data were corrected for dead-time overlap by solving the system of equations (16) described in the Methods section “Spencer’s integral method”. This process was repeated for ten different dead-times. For each dead-time value, $10^3$ MC examples were used to test Spencer’s method. The distribution of errors for each multiplicity probability was fit with a Gaussian function and the mean value with standard deviation for multiplicities 3, 4, 5 and 6 are shown in Fig. 5. The results clearly show a mild degeneracy growing with dead-time impacting the higher multiplicities more than the lower ones. Therefore, the analytical description provided by this method despite being more accurate than Boldeman’s method, do not reflect the whole complexity of the problem, and again the solution of the system of equations become degenerate. The fractional errors for multiplicities 3, 4, 5 and 6 are around ± 7%, ± 7%, ± 13%, and ± 60% respectively at $\tau$ = 0.9 $\upmu$s.

The errors observed in Fig. 1 generate a 0.9% mean error in the average number of neutrons per detection gate calculated with ${\bar{\nu }}=\sum n D_n$. The average number of neutrons emitted per detection gate is 3.757 for $^{252}$Cf from the work of Santi^17^ and 3.791(7) if using Spencer’s method. This result seems to indicate that the degeneracy observed in Spencer’s method do not affect ${\bar{\nu }}$ and is somehow compensated. This compensation ends up masking the errors of this method if one is only interested in determining ${\bar{\nu }}$.

### Moat’s MC method

**Figure 6:**
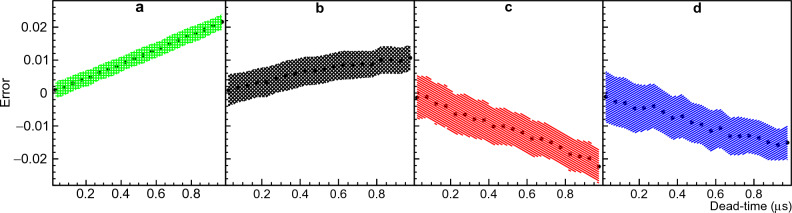
Errors from Moat’s method. The mean value and standard deviation resulting from the Gaussian fit of the error distribution for twenty different dead-times was plotted. Panels (**a**), (**b**), (**c**), and (**d**) correspond to neutron multiplicities 2, 3, 4, and 5, respectively. For more details please refer to text.

Moat’s method is described in the Methods section “Moat’s MC method”. Similar to the other methods, a MC-generated data was used to test Moat’s method. The method was tested for different dead-times in the range 0.05 to 1.0 $\upmu$s. The error distribution of each neutron multiplicity probability was fit with a Gaussian function. The mean value and standard deviation of the Gaussian fit for multiplicities 2, 3, 4 and 5 are shown in Fig. 6. The mean fractional errors for multiplicities 2, 3, 4, and 5 are around 18%, 4%, $-8\%$, and $-9\%$ respectively at $\tau$ = 0.9 $\upmu$s.

The results of Moat’s method do not present degeneracy as the results of Boldeman’s and Spencer’s methods. However, the errors of the neutron multiplicity probabilities from the true values grow linearly as a function of the dead-time. These trends show that the corrected distribution is being shifted to the left as the dead-time gets longer. The error of multiplicities 2 and 3 get more negative because they are on the left side of the peak center and the error in multiplicities 4 and 5 get more positive because they are on the right side of the peak center. If the multiplicity distribution is shifting to the left, this means that the correction is smaller than needed and the method is missing something.

The errors observed in Fig. 1 generate a 2.4% error in the average number of neutrons per detection gate calculated with ${\bar{\nu }}=\sum n D_n$. The average number of neutrons emitted per detection gate is 3.757 for $^{252}$Cf from the work of Santi^17^ and 3.667(5) if using Moat’s method.

### The NN method

**Figure 7:**
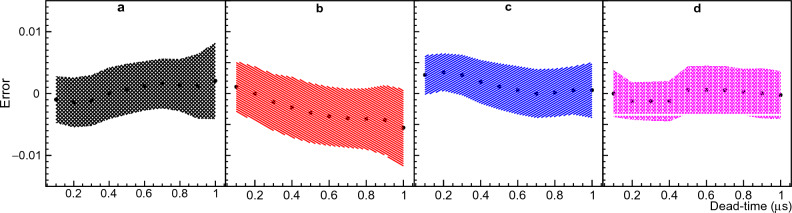
Errors from NN’s method. The mean value and standard deviation resulting from the Gaussian fit of the error distribution for ten different dead-times was plotted. Panels (**a**), (**b**), (**c**), and (**d**) correspond to neutron multiplicities 3, 4, 5, and 6, respectively. For more details please refer to text.

The NN method proposed in this work is described in the Methods section “The NN method”. The MC-generated data was used to test the NN method for different dead-times in the range 0.05 to 1.0 $\upmu$s. The error distribution of each neutron multiplicity probability was fit with a Gaussian function. The mean value and standard deviation of the Gaussian fit for multiplicities 3, 4, 5 and 6 are shown in Fig. 7. The fractional errors for multiplicities 3, 4, 5 and 6 are around ± 1.5%, 3%, ± 2%, and ± 5% respectively at $\tau$ = 0.9 $\upmu$s.

The NN was trained with Gaussian and skewed Gaussian multiplicity probability distributions with different shapes covering a broad range of possible distributions with different mean values. Due to the random initialization of weights and the random division of training and testing examples, multiple networks can be obtained for the same training dataset. To account for this statistical variation, we trained the NN 100 times, resulting in an ensemble of 100 NNs. For each multiplicity, the probability distribution obtained from the ensemble was fit with a Gaussian function. The mean value and standard deviation of each Gaussian fit are shown in Fig. 1, along with the true neutron multiplicity probability distribution of $^{252}$Cf from the work of Santi^17^ and the error from the true values.

The mean errors observed in Fig. 1 are consistently smaller than 0.005, equivalent to fractional errors smaller than 2% for multiplicities 3 and 5, and generate a 0.5% error in the average number of neutrons per detection gate calculated with ${\bar{\nu }}=\sum n D_n$. The average number of neutrons emitted per detection gate is 3.757 in the case of $^{252}$Cf from the work of Santi^17^ and 3.775(49) if using the NN method. Notably, the neural network exhibits a higher degree of resilience toward statistical variations when compared to both Boldeman’s and Moat’s methods. This finding highlights a key advantage of using NNs, namely the ability to generalize. The effectiveness of NN generalization relies heavily on the quality and diversity of its training data. The training data must include all possible expected multiplicity distributions. In the case of our training dataset, it included all possible Gaussian and skewed Gaussian distributions with perturbations of ±10% for each multiplicity probability in the studied multiplicity range. It also included narrow Gaussians which is the equivalent of a single multiplicity. Therefore, if the expected multiplicity distribution differs from a Gaussian, or skewed Gaussian, e.g. two peaks, the neural network may fail in unfolding it. This problem can be easily solved by training the neural network with distributions like the expected. This increases the robustness of the method since the training data comes from Monte Carlo simulations that can be easily multiplied.

## Conclusion

This work demonstrates the successful application of a method using neural networks for dead-time correction and background subtraction for detectors with time-dependent neutron capture. In this work, we reviewed and tested three other methods available in the literature for different dead-times. While Boldeman’s analytical method proved effective for dead-times below 0.2 $\upmu$s, for larger dead-times it becomes degenerate, resulting in fractional errors of more than 100% for all multiplicities. Spencer’s integral method demonstrates a mild degeneracy that grows with dead-time resulting in fractional errors of more than 7% for all multiplicities. Although Moat’s method yields a more accurate result compared to Boldeman’s, it shifts the multiplicity distribution towards lower multiplicities as the dead-time gets longer. The NN method is more accurate and precise than any of the three methods studied in this work. An example is given using the neutron multiplicity probability distribution of $^{252}$Cf, where the average number of neutrons per detection gate resulting from Moat’s method has an error of 2.4% from the true value while the result from the NN method has an error of 0.5% from the true value. In fact, the principle of the NN method described in this work can be applied to any physics problem where one is confident that the system studied can be simulated without being able to analytically describe the system accurately. In addition, the NN method can be easily modified if multiplicities greater than 10 or if multiplicity distributions different from gaussian and skewed gaussians are expected. In this paper, we have developed and successfully demonstrated a novel approach using neural networks trained with data from a MC model that simulates the dead-time and background effects. The neural network corrects the measured neutron multiplicity probability distribution for background and dead-time overlap all at once. This approach can now be broadly applied to all experiments measuring multiplicity probabilities utilizing detectors with time-dependent events in the detection gate.

## Methods

### Time distribution of neutrons captured in gadolinium-loaded liquid scintillators

The dead-time correction depends primarily on the neutron capture time distribution. Therefore, it is important to understand this process and the equation describing the time distribution. To be absorbed by a gadolinium loaded liquid scintillator the neutron needs to be moderated to a thermal energy (G) typically on the order of 0.05 to 0.1 eV^12^. The probability that a neutron moderated to an energy G becomes absorbed in the time range ($t'$, $t'$+$dt'$) is given by equation (1), where $t'$ is the time measured from the trigger reference point and $dt'$ is an infinitesimal increment of time. The reference point can be the fission fragments in the case of a fissioning nucleus, or the prompt $\gamma$-rays coming from a nuclear reaction^21^.

1 $$\begin{aligned} k h(t')dt' = k dt' \int _{0}^{t'} \beta g(t) \,e^{-\beta (t'-t)}dt , \end{aligned}$$

where k is the probability that the neutron will eventually be captured in the gadolinium, $\beta$ is a “slowing down” parameter which depends mainly on the actual amount of the loading material present in the liquid scintillator and *g*(*t*) is a probability density representing the neutron thermalization time and subsequent capture time of the form

2 $$\begin{aligned} g(t) = \lambda ^2 t \,e^{-\lambda t} , \end{aligned}$$

where the reciprocal of $\lambda$ is a “slowing down” parameter, with 2/$\lambda$ being the mean time for neutrons to become moderated to G, and *g*(*t*)*dt* is the probability that a neutron is moderated to energy G in a time interval (*t*, $t+dt$). Substituting eq. 2 into eq. 1 gives

3 $$\begin{aligned} h(t') = \beta \lambda ^2 \,e^{-\beta t'} \int _{0}^{t'} t \,e^{(\beta -\lambda )t}dt . \end{aligned}$$

After solving the integral in equation (3) we obtain

4 $$\begin{aligned} h(t') = \frac{\beta \lambda ^2}{(\beta - \lambda )^2} (\,e^{\lambda t'}[(\beta -\lambda )t'-1] + \,e^{-\beta t'}) . \end{aligned}$$

**Figure 8:**
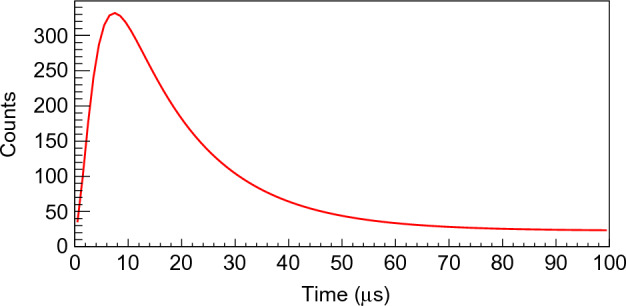
Neutron capture time distribution. The neutron capture time distribution for a gadolinium loaded liquid scintillator was obtained by fitting the experimental time distribution reported by Akindele^16^ using equation (4).

The resulting curve from fitting the time distribution measured by Akindele^16^ using equation (4) is shown in Fig. 8. Equation (4) gives the best analytical representation of the capture time distribution in a gadolinium loaded liquid scintillator. If a neutron detector with different capture element has a different time dependent capture distribution e.g. Poisson, Gaussian or any other, the results may differ a little bit depending on the resulting overlap probability, which is what mostly affects our results. The overlap probability depends on the integral of the square of the the normalized capture time distribution. Therefore, the smaller the width of the time distribution, the larger the overlap probability. Our results demonstrate that the larger the overlap probability, the worst all available methods perform.

Finally, the normalized time distribution *f*(*t*) is given by

5 $$\begin{aligned} f(t) = h(t) \left[ \int _{0}^{T} h(t') dt' \right] ^{-1} , \end{aligned}$$

where *T* is the detection gate time length.

### Boldeman’s analytical method

The dead-time correction for neutron multiplicity counters introduced by Dytlewski^22^ and further expanded by Croft^23^ does not apply to a liquid scintillator because the neutron capture temporal distribution of a liquid scintillator is not constant in time. The equations to correct the neutron multiplicity probability distribution for a single overlap per detection gate in Boldeman’s method are described in the work of Boldeman^8,9^.

If $F'_{l}$ is the probability per reaction of recording *l* pulses during the neutron counting gate, $B'_x$ the probability of recording *x* background pulses during the background counting gate, $D_x$ and $B_x$ are the real probabilities of occurrence of x neutron pulses during the neutron counting gate and x background pulses during the background counting gate respectively, the following equations may be written as

6 $$\begin{aligned} F'_l= & {} \sum _{x=0}^{l} D_x B_{l-x} \left[ 1 - \, ^{x} \, C_2 \, k_{nn} - x(l-x)k_{nb} - \, ^{(l-x)} \, C_2 \, k_{bb} \right] + \sum _{x=0}^{l+1} D_x B_{l+1-x} \left[ ^{x} \, C_2 \, k_{nn} + x (l+1-x) k_{nb} + \, ^{(l+1-x)} \, C_2 \, k_{bb} \right] , \end{aligned}$$

7 $$\begin{aligned} B'_x= & {} B_x (1 - \, ^{x} \, C_2 \, k_{bb}) + B_{x+1} \, ^{(x+1)} \, C_2 \, k_{bb} , \end{aligned}$$

where $k_{nn}$ is the probability that two neutron pulses, occurring during the neutron counting gate, overlap and appear as one pulse; $k_{nb}$ is the probability that one neutron and one background pulse overlap; $k_{bb}$ is the probability that two background pulses overlap; and, $^{X} \, C_Y$ is the combination probability of selecting Y items out of X^2,10^,

8 $$\begin{aligned} ^{X} \, C_Y = \left( {\begin{array}{c}X\\ Y\end{array}}\right) = \frac{X!}{Y!(X-Y)!} . \end{aligned}$$

The overlap probabilities can be written as

9 $$\begin{aligned} k_{nn}= & {} 2 \tau \int _{0}^{T} f^2(t) dt , \end{aligned}$$

10 $$\begin{aligned} k_{nb}= & {} 2 \tau \int _{0}^{T} \frac{f(t)}{T} dt = \frac{2\tau }{T} , \end{aligned}$$

11 $$\begin{aligned} k_{bb}= & {} \frac{2\tau }{T} , \end{aligned}$$

where $\tau$ is the dead-time, and T is the duration of the detection gate.

Note that equations (6) and (7) account only for the probability of a single overlap per detection gate. The dead-time measured by Boldeman^9^ is approximately 70 ns. Since the dead-time for the NeutronSTARS detector is more than ten times longer, we extended equations (6) and (7) to include two overlaps per detection gate:

12 $$\begin{aligned} \begin{aligned} F'_l = \sum _{x=0}^{l}&D_x B_{l-x} [1 - \, ^{x} \, C_2 \, k_{nn} - x(l-x)k_{nb} - \, ^{(l-x)} \, C_2 \, k_{bb} - \, ^{x} \, C_3 k_{nnn} -^{x} \, C_2 \, (l-x) k_{nnb} - \, ^{(l-x)} \, C_2 \, x k_{nbb} -^{(l-x)} \, C_3 k_{bbb} \\&- \, ^{x} \, C_4 k_{2nn} - \, ^{x} \, C_2 \, \, ^{(l-x)} \, C_2 \, k_{2nb} - \, ^{x} \, C_3 (l-x) k_{nn,nb} - \, ^{(l-x)} \, C_3 x k_{nb,bb} -^{(l-x)} \, C_4 k_{2bb}] \\ + \sum _{x=0}^{l+1}&D_x B_{l+1-x}[^{x} \, C_2 \, k_{nn} + x(l+1-x)k_{nb} + \, ^{(l+1-x)} \, C_2 \, k_{bb}] \\ + \sum _{x=0}^{l+2}&D_x B_{l+2-x}[^{x} \, C_3 k_{nnn} + \, ^{x} \, C_2 \, x(l+2-x)k_{nnb}+ \, ^{(l+2-x)} \, C_2 \, x k_{nbb} + \, ^{(l+2-x)} \, C_3 k_{bbb} \\&+ \, ^{x} \, C_4 k_{2nn} + \, ^{x} \, C_2 \, \, ^{(l+2-x)} \, C_2 \, k_{2nb} + \, ^{x} \, C_3 (l+2-x) k_{nn,nb} + \, ^{(l+2-x)} \, C_4 k_{2bb}] \end{aligned} \end{aligned}$$

13 $$\begin{aligned} B'_x = B_x (1 - \, ^{x} \, C_2 \, k_{bb} - \, ^{x} \, C_3 k_{bbb} - \, ^{x} \, C_4 k_{2bb}) + B_{x+1} \, ^{(x+1)} \, C_2 \, k_{bb} + B_{x+2} \, ^{(x+2)} \, C_3 k_{bbb} + B_{x+2} \, ^{(x+2)} \, C_4 k_{2bb} \end{aligned}$$

where the overlap probabilities $k_{nnn} = k_{nn}^2$, $k_{nnb} = \frac{3}{4} k_{nn} k_{nb}$, $k_{nbb} = \frac{3}{4} k_{nb} k_{bb}$, and $k_{bbb} = \frac{3}{4} k_{bb}^2$ are the probabilities per detection gate of one event overlapping with two other events. The overlap probabilities $k_{2nn} = k_{nn}^2$ $^{4} \, C_2/2!$, $k_{2nb} = k_{nb}^2$ $^{4} \, C_2/2!$, $k_{2bb} = k_{bb}^2$ $^{4} \, C_2/2!$, $k_{nn,nb} = k_{nn} k_{nb}$ $^{4} \, C_2/2!$, and $k_{nb,bb} = k_{nb} k_{bb}$ $^{4} \, C_2/2!$ are the probabilities of two separate overlaps.

#### Implementation of Boldeman’s method

Equations (12) and (13) were implemented in C++ as an array of functions set to zero. The implementation consisted of 20 $B'_x$ functions and 15 $F'_l$ functions covering the intervals $B_0$ to $B_{19}$ and $D_0$ to $D_{14}$. In order to ensure the number of equations is equal to the number of variables, adjustments were made to the last but one equation of $B'_x$ and $F'_l$. Specifically, the multiplicity probabilities $D_{15}$ and $B_{20}$ were assumed to be zero and were removed from both system of equations. Additionally, in the last equation of $B'_x$ and $F'_l$ systems of equations, the terms containing $D_{15}$, $B_{20}$, $D_{16}$ and $B_{21}$ were omitted. Indeed, the multiplicity probabilities $D_{15}$ and $D_{16}$ are zero in the case of $^{252}$Cf as shown in Fig. 1, and the multiplicity probabilities $B_{20}$ and $B_{21}$ are zero for a background rate of 20 kHz in a detection window of 60 $\upmu$s.

The two systems of equations, $B'_x$ and $F'_l$, are solved independently using ROOT^24^. The first system, $B'_x$, is solved to obtain the probabilities $B_x$, while the second system, $F'_l$, is solved to obtain the probabilities $D_x$. Each system of equations is wrapped using *ROOT::Math::WrappedParamFunction* with the corresponding parameters: $B'_x$, $F'_l$, the overlap probabilities, and the probabilities $B_x$ obtained from the $B'_x$ system of equations. The wrapped functions are then added to a *ROOT::Math::GSLMultiRootFinder* object, enabling the separate solution of each system of equations for the probabilities $B_x$ and $D_x$.

#### Monte Carlo model to test Boldeman’s method

The experimentally measured multiplicity probabilities $B'_x$, $F'_l$ and the overlap probabilities were generated with a MC model that simulates the data acquisition process including the background and the dead-time overlap effects in the measured multiplicity probability distribution. The MC receives as inputs the real multiplicity probability distribution, the time distribution of neutron capture, the background rate, the detection gate duration and the dead-time.

Analogous to the section “dead-time overlap effect”, the neutron multiplicity was sampled from the standard neutron multiplicity probability distribution of $^{252}$Cf from the work of Santi^17^, and the time of each event was sampled from the time distribution shown in Fig. 8. A time difference matrix was then generated for each given detection window with the time difference between all available neutron and background events. The dead-time overlap followed a simple selection rule where if the time difference is smaller than $\tau$ = 0.9 $\upmu$s, the event with a longer time was rejected. Each iteration corresponds to a detection gate and is referred to as a “run”. A set of $10^5$ runs is considered as one output containing the neutron multiplicity distribution simulating the experimental distribution. By performing the Monte Carlo simulation $10^3$ times, we generated $10^3$ outputs-equivalent to $10^3$ simulated experimental distributions, each sharing identical input parameters. The simulated data were then given to the solver for Boldeman’s method. The results from the solver for each individual multiplicity had a distribution due to the MC statistical variation. Each multiplicity was fit with a Gaussian function, and the mean value and standard deviation of the resulting fit were recorded. These were the inputs used in the Monte Carlo model: Multiplicity probability distribution of $^{252}$Cf reported by Santi^17^; Time distribution of neutron capture measured by Akindele^16^; Background rate of 20 kHz from previous experiments using NeutronSTARS; Detection gate length of 60 $\upmu$s; and, dead-time of 0.9 $\upmu$s.

### Spencer’s integral method

In Spencer’s work^13^, the neutron multiplicity probability distribution is first corrected for the background and then for the dead-time overlap with an analytical method.

Spencer’s systems of equations were solved using the same procedure explained in the Methods section “Implementation of Boldeman’s method”. The MC-generated data to test Spencer’s method were obtained with the MC model described in the Methods section “Monte Carlo model to test Boldeman’s method”.

#### Background correction

The background corrected neutron multiplicity probability distribution, $N_n$, is obtained by solving the following equations,

14 $$\begin{aligned} F'_n = \sum _{i=0}^{n} B_{n-i,i} \, \, N_i , \end{aligned}$$

where $F'_n$ is the measured neutron multiplicity probability distribution, $B_{n,i}$ is the probability distribution of *n* background events occurring in the detection window when *i* neutrons are present,

15 $$\begin{aligned} B_{n,i} = B'_n \left( 1-\frac{2ni\tau }{T} \right) + B'_{n+1} \frac{2(n+1)i\tau }{T} , \end{aligned}$$

and $B_n$ is the measured background multiplicity probability distribution. In the present work, we considered the multiplicity range from 0 to 10. This system of equations was solved using the same solver implemented described in Boldeman’s method.

#### dead-time overlap correction

The dead-time corrected multiplicity probability distribution, $D_j$, is obtained by solving the following equations,

16 $$\begin{aligned} N_i = \sum _{j=i}^{j_{max}} S_{i,j} \, D_j , \end{aligned}$$

where $N_i$ is the background corrected neutron multiplicity probability distribution, and $S_{i,j}$ represents the probability that only *i* neutrons will be counted when *j* neutrons are actually present within the detection window,

17 $$\begin{aligned} S_{i,j} = \frac{j!}{(j-i)!} \int \limits _{0}^{T-(i-1)\tau } \hspace{-10.0pt}dt_1 \, f(t_1) \, \int \limits _{t_1 + \tau }^{T-(i-2)\tau } \hspace{-10.0pt}dt_2 \, f(t_2) \, \ldots \, \int \limits _{t_{i-1}+\tau }^{T} \hspace{-5.55542pt}dt_i \, f(t_i) \, G_{i,j} (t_1, t_2, \ldots , t_i) , \end{aligned}$$

where *f*(*t*) is the neutron capture time distribution of Fig. 8, and the function $G_{i,j}=1$ if $i=j$, $G_{i,j}=0$ if $i>j$, and for $i<j$,

18 $$\begin{aligned} G_{i,j}(t_1, t_2, \ldots , t_i) = \left( \sum _{k=1}^{i} \, P_{k} \, \right) ^{j-i} , \end{aligned}$$

where

19 $$\begin{aligned} P_k = \int \limits _{t_k}^{t_k+\tau } dt' \, f(t') , \, k = 1 \ldots i. \end{aligned}$$

The multidimensional integral in $S_{i,j}$ was solved numerically using the MC integration algorithm VEGAS^25^ implemented in the ROOT class *ROOT::Math::GSLMCIntegrator*. To implement the multidimensional numerical integration in $S_{i,j}$, variables $t_2 \ldots t_i$ were substituted, setting the new integration limits from 0 to 1. The relative error tolerance of each matrix element was fixed to $10^{-5}$, i.e. 0.01%.

### Moat’s MC method

Moat’s method^4^ involves obtaining the coefficients of the matrix ${\varvec{{T}}}$ that transforms ${\varvec{{D}}} =\{D_0,...,D_l\}$ into ${\varvec{{F}}}' =\{F'_0,...,F'_l\}$ through a MC simulation. The coefficients *T*$_{mn}$ of the transformation matrix are defined as

20 $$\begin{aligned} T_{mn} = \sum _{j=m-n}^{J} B_j \, k_m(n,j) , \end{aligned}$$

where *j* starts from $m-n$ for $m\ge n$, and for $m < n$ it starts from 0. The value of *J* adopted for each summation is such that $B_{(J+1)}$ is near zero. The probabilities $B_j$ are the background multiplicity probabilities after dead-time correction and can be obtained by solving the system of equations from equation (13). The term $k_m(n,j)$ represents the probability of observing *m* pulses when *n* neutron pulses are produced according to the scintillator capture time distribution and *j* background pulses are produced with uniform probability distribution in the detection gate. The $k_m(n,j)$ probabilities include dead-time overlaps and can be calculated using the MC model described in the Methods section “Monte Carlo model to test Boldeman’s method” with small modifications to the code. The system of equations obtained from ${\varvec{{F}}}' = {\varvec{{T}}} {\varvec{{D}}}$ can be written as

21 $$\begin{aligned} F'_m = \sum _{n=0}^{l} T_{mn} D_n , \end{aligned}$$

where $F'_m$ are the experimentally measured multiplicity probabilities and $D_n$ are the real neutron multiplicity probabilities.

The $k_m(n,j)$ probabilities were calculated after 10$^8$ runs with the neutron and background multiplicities ranging from 0 to 10 for twenty different dead-times. For each set of $k_m(n,j)$ probabilities associated with a dead-time, the $F'_m$ and $B_j$ multiplicity probabilities were obtained after 10$^5$ runs of the MC model described in the Methods section “Monte Carlo model to test Boldeman’s method”.

### The NN method

To enhance the accuracy of the results and overcome the dead-time limitation, we employed a NN trained with Monte Carlo data. Traditionally, NNs are trained using Python, but our existing codes were in C++. Therefore, we investigated the availability of a NN framework within the ROOT software package, which is based on C++, aligning with our coding language. We used the Deep Learning module in the TMVA (Toolkit for Multivariate Data Analysis) toolkit, which offers a deep learning architecture called Deep Neural Network (DNN). The TMVA/DNN architecture provides an optimized implementation of feed-forward multilayer perceptrons. It has distinct advantages, such as the ability to utilize multi-core and GPU hardware architectures and various optimization methods such as momentum-based learning^26^. To construct our NN, we referred to the examples “TMVARegression.C” and “TMVARegressionApplication.C”^26^. These steps were followed for training the NN:

1. Eleven $F'_l$ variables and one dead-time variable are declared for the input neuron layer, and eleven $D_x$ targets are declared for the output neuron layer.
2. The dataset tree is loaded and randomly split into training and testing trees, each containing half of the examples from the training dataset.
3. The NN is defined with three hidden layers, each with 50 neurons (12/50/50/50/11). All neurons in the network use the hyperbolic tangent activation function (TANH).
4. The optimization method ADAM^27^ is used, with common parameter values^26,28^. The learning rate is set to 10$^{-4}$, the momentum to 0.9, the convergence steps to 10, the batch size to 128, the weight decay to 10$^{-4}$, and beta1 = 0.9, beta2 = 0.999, and eps = 10$^{-7}$.
5. The weights are initialized using the XAVIER method, which randomly initializes the connection weights of each layer.
6. The network is trained until the learning rate does not improve after 10 steps;
7. The weights obtained are saved in a file to later test the network with the testing datasets.

The training dataset is composed of example sets of $\tau$, $F'_0$...$F'_{10}$ and $D_0$...$D_{10}$ where the dead-time $\tau$ and the probabilities $F'_l$ are the inputs and the probabilities $D_l$ the target values. Each example set had a different initial input multiplicity probability distribution $D_l$. For each set of the probabilities $D_l$, the resulting probabilities $F'_l$ were obtained using the MC model described in the Methods section “Monte Carlo model to test Boldeman’s method”. We trained the NN using three distinct datasets: one comprising solely of Gaussian multiplicity probability distributions, another consisting exclusively of skewed Gaussian multiplicity probability distributions, and a third dataset that combined both Gaussian and skewed Gaussian multiplicity probability distributions, all with around 2$\times$10$^6$ examples. The NNs trained on the first two datasets yielded errors of up to 0.03, while the NN trained on the composite dataset exhibited errors smaller than 0.01. In addition, we generated a fourth dataset comprising Gaussian and skewed Gaussian multiplicity probability distributions with ±10% perturbations for each multiplicity probability in the whole dead-time range from 0 to 1.1 $\upmu$s. The NN trained in the fourth dataset containing around 2$\times$10$^6$ examples yielded errors smaller than 0.005. Therefore, we show the results for the NNs trained in the fourth perturbed composite dataset which had mean values randomly distributed in the interval [1, 9], standard deviation randomly distributed in the interval [0.1, 3.0], and skewness randomly distributed in the interval $[-2, 2]$.

## Data Availability

The datasets generated and analyzed during the current study are available from the corresponding author on reasonable request.
